# Statistical Study of Risk Factors of End Stage Renal Failure in Peshawar, Pakistan

**DOI:** 10.3889/oamjms.2015.009

**Published:** 2015-03-05

**Authors:** Salahuddin Khan, Tariq Hussain, Hashimuddin Azam, Najma Salahuddin

**Affiliations:** 1 *Department of Family and Community Medicine, College of Medicine, University of Dammam, Kingdom of Saudi Arabia*; 2 *Higher Education Department, Peshawar, Pakistan*; 3 *Department of Medicine, Khyber Medical College, Peshawar, Pakistan*; 4 *Department of Statistics, Shaheed Benazir Bhutto Women University, Peshawar, Pakistan*

**Keywords:** End Stage Renal Failure, Logistic regression, Backward elimination, Brown test, Risk Factors

## Abstract

**BACKGROUND:**

End Stage Renal Failure (ESRD) is the last stage of the chronic renal failure in which kidneys become completely fail to function.

**AIM:**

The basic aim of this study was to discover the important risk factors of ESRD and to construct a model for prediction of the ESRD patients in various hospitals of Peshawar, Pakistan.

**MATERIAL AND METHODS:**

The data were collected from the patients of renal diseases from three major hospitals of Peshawar. Brown method was used to obtain initial model, then backward elimination logistic regression analysis was performed to find the significant variables (risk factors). The response variable (ESRD) in this study is binary; therefore, logistic regression analysis is used to identify the significant variables. A Statistical Package GLIM and SPSS were used for fitting the model and for finding the significant variables.

**RESULTS:**

The backward elimination procedure selects predictor variables diabetic, hypertension, glomerulonephritis and heredity, for males. Thus, these variables are the main causes of ESRD. For females, the predictor variables selected are hypertension & (Diabetic*Hypertension), which means that hypertension and hypertensive diabetic are significant causes of ESRD.

**CONCLUSION:**

Our main conclusion from this analysis is that diabetic, hypertension and glomerulonephritis are the significant risk factors of ESRD.

## Introduction

Statistical science consists of ever growing collection techniques used to make wise decisions in the face of uncertainty. Statistical methods are integral part of research in almost all fields of study. The branch of statistics which study the data about the living organism and is very much an indispensable tool in the field of medical research and its development is called Biostatistics. In carrying out medical research programs, one cannot deny biostatistics to be the integral part of it. Statistical methods are used in finding significant risk factor of various diseases and also applicable to psychology, taxonomy etc. “In statistics, the mathematical modeling plays very important role, specially probability theory which is used to narrate the variation in the data and as a source of drawing conclusions about them” [1].

The generalized linear models are extensively used in the social science and especially in medical research, introduced by Nelder and Wedderburn [2]. It is beneficial to use generalized linear model for evaluating the significant risk factors of end stage renal disease.

The main function of kidney is to filter the blood purified and making the waste product to go out of the body. Kidney also adjusts the blood pressure by assessment of electrolytes in the body and stimulating the production of red blood cells. Kidney diseases are divided into two groups: the first one is acute renal disease and the second one is chronic renal disease. In medical science the term kidney disease is known as renal disease.

### End Stage Renal Diseases (ESRD)

ESRD is the last stage of the chronic renal failure in which kidneys become completely fail to function. In this stage the kidney stop its functions to remove the impurities and control electrolytes. The symptoms of ESRD comprise less urine output, swelling of legs, face, nausea and vomiting [3].

A cohort study was carried out by Hsu and colleagues in 2009. By using the statistical technique of survival analysis, it was found that the independent risk factors of ESRD were sex, race, anemia and heredity [4]. A research of 600 people was carried out by using the Odds Ratio analysis and it was found that diabetes has strong association with ESRD. The systolic and diastolic hypertension accelerates diabetic nephropathy. Smoking also increases the risk of ESRD in diabetes patients [5].

In a cohort study, it was found that kidney failure was more common in the United States likely; reflecting an aging population with increased rates of hypertension, diabetes and obesity and that the Cardio Vascular Disease (CVD) was a major risk factor of ESRD [6]. The researcher also found an interesting risk factor, body mass index which is associated with ESRD and it has a j-shape relationship with ESRD [6].

Kamal and Sakhuja [7] proposed that the key source of ESRD in Pakistan and India are hypertension, glomerulonephritis and diabetes. One third of the ESRD patients are affected by glomerulonephritis, while diabetic nephropathy accounts for about one fourth of all patients in India. The average age of ESRD in the subcontinent is lesser (42 years) than the western countries (61 years).

It was found that the leading risk factors of ESRD are glomerulonephritis, diabetes, hypertension, and polycystic kidney disease [8]. In a study of kidney patients, the researchers found that diabetes was the major cause of ESRD and were 44.603 % of the total ESRD patients, whereas hypertension was 26.587 % and glomerulonephritis was 12.19 % of the total ESRD patients. Further, 16 % of the total ESRD patients were affected by Systemic Lupus Erythematous, Obstructive Nephropathy etc. Some other risk factors (such as age, sex, race and family history) were also caused for the growth of ESRD [8].

The basic aim of this study was to discover the important risk factors of ESRD and to construct a model for prediction of the ESRD patients in various hospitals of Peshawar, Pakistan.

## Materials and Method

The circumstance challenging the researcher in medical studies is the enlightening of significant predictive aspects that disturb various outcomes of interest. The researcher might, for example, be interested in finding in what way response to a stroke patient is influenced by diabetes and age. The statistical device for such a status quo is some form of regression model. Regression analysis is a statistical method used to examine the affiliation flanked by dependent and different independent variables. In several cases, the persistence of the analysis is prediction, in others it is simply to determine vital predictive variable. Different kinds of regression methods are available, analogous to the different natures of explained variable. Simple linear regression and multiple regressions, for example, are applicable for continuous explained variables such as blood pressure or weight. Logistic regression is appropriate to states concerning binary variables, for example, existence or nonexistence of ESRD, alive or dead at the end of a clinical trial, gets better or not from a disease.

In this study, we examine the relationship of ESRD (a binary response variable) with various risk factors: age, sex, diabetes, hypertension, glomerulonephritis, hepatitis, heredity, SLE nephritis, polycystic kidney disease, heart disease and obstructive nephropathy. So the precise instrument is the logistic regression modeling.

### Data Collection

To identify the important risk factors of ESRD, the data were collected from three main hospitals of Peshawar namely Hayatabad Medical Complex, Khyber Teaching Hospital and Lady Reading Hospital. A total of 407 cases were examined for the presence or absence of ESRD. The risk factors for this study were diabetes, hypertension, glomerulonephritis, polycystic kidney diseases, obstructive nephropathy, myeloma, SLE nephritis, hepatitis, family history, drug usage, heart problem, age and sex. The information was collected for patients that belong to uncontrolled group (ESRD cases) and also to control group (non-ESRD cases). There were 407 patients in which 244 (60%) were males and 163 (40%) were females. The average age of male patients was 43.38 years and females patients average age was 42.4 years. Out of 407 patients 167 had ESRD and 240 had no ESRD. Of 244 male’s patients, 108 patients had ESRD and 136 had no ESRD. Of 163 female patients 59 had ESRD and 104 had no ESRD, which indicate that males are more exposed to ESRD as compared to females.

### Logistic Regression Modeling

Berkson [9] suggested the logistic regression model at first and told how we fit the model by iteratively weighted least squares. In medical studies, the logistic regression is commonly used; subsequently numerous studies contain two-category response or explained variables. An early example of the use of this method, in the situation of a study of 12-year prevalence of coronary heart disease, was discussed [10]. Seven different factors measured at initial examination were studied for their effects on the incidence of the disease; they were age (in years), ECG (normal or abnormal), systolic blood pressure (mm Hg), cigarettes smoked per day relative weight, hemoglobin (g/dl), and serum cholesterol (mg/dl). Age and cholesterol level were found to be the top significant predictive variables [10].

The relationship of a subject’s health status with age, smoking behavior, sex and socioeconomic group was studied [11]. The main purpose of the analysis was to conclude whether chronic sickness was more prevalent in smokers after modification for gender, age and socioeconomic group [11]. An outstanding behavior of logistic regression is that of Cox and Snell [12].

One form of the multiple linear regression models is:

Y = β_0_ + ∑ β_i_X_i_ + ε

E (Y) = β_0_ + β_1_X_1_ + β_2_X_2_ + …+ β_k_X_k_ (1)

E (Y) = β_0_ + ∑ β_i_X_i_

where Y is the response variable and is equal to 1, if patients has an ESRD, otherwise 0 (for non-ESRD patients). The E(Y) in equation (1) is consider to be, any real value except we restrain the values of β_0,_ β_1_, β_2_, …, β_k_. The E(Y), where Y is binary, approaches the probability that Y=1, that is, E (Y) = 1, P [Y=l] + P[Y=O] =P[Y=1].

Let us denote this probability by P(x), reflecting its dependence on the independent variables X_1_ + X_2_ + …+ X_k_. Since E (Y^2^) = 1^2^ P(x) + 0^2^ P [1 – P (x)] = P (x), therefore, the Var(Y) is given as: = *var*(*Y*) = *E*[*Y* − *E*(*Y*)]^2^ = *E*(*Y*^2^) − [*E*(*Y*)]^2^ = *P*(*x*).*P*[1−*p*(*x*)]. Hence if we write “P” instead of P(x), for the sack of simplicity, then the equation (1) can be written as:

E (Y) = P = β_0_X_0_ + β_1_X_1_ + β_2_X_2_ + …+ β_k_ X_k._ (2)

For dichotomous explained variable Y, the equation (2) is known as linear probability model. When observations on Y are independent, this model is said to be Generalized Linear Model with identity link function. It is better to study models by using a curvilinear link flank by X & P due to structural problem related with the equation (2). After alteration, it is better to assume the linear model for the dependent variable. Logistic or Logit is the common way for transformation of equation (2), which can be expressed mathematically as:


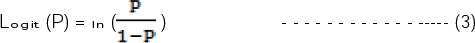


where 0 < P < 1. Now the required model is:


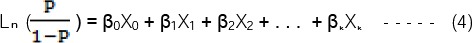


The linear probability model in equation (2) can now be re-written as:


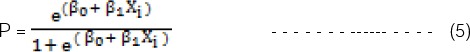


For a particular X, the equation (5) becomes,


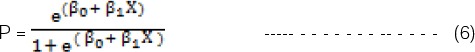


Generalized Linear Model is the link function for the logistic regression. For this model, the odds of making response are:


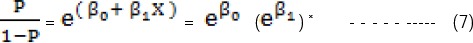



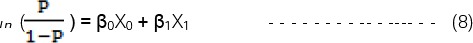


If one unit increases in X, then the odds **e^β_1_^** increase times. There is linear association in the Log odds for which suitable way is the Logit that is, the transformation of log odds. If the sampling is prospective or retrospective, the effect can be estimated by this model. The odds are the influence of the logistic model and the ratio of odds of one observation to the odds of other is known as odds ratio.

### Analysis

The data were collected from the patients of renal diseases from three major hospitals of Peshawar namely Hayatabad Medical Complex, Khyber Teaching Hospital and Lady Reading Hospital. A total of 407 cases were examined for the presence or absence of ESRD. For this study, a complete history of patients were taken which include Sex (S), Diabetes (D), Hypertension (H), Obstructive Nephropathy (O), Glomerulonephritis (G), Polycystic kidney diseases (Pk), Myeloma (M), SLE nephritis (SLE), Anemia (A), Heredity (Hd), Hepatitis (Ht), Excess use of drug (Ed) and Heart problem (Hp), while the ESRD was observed as response variable which are categorical variables. For this purpose, Brown method was used to obtain initial model, then backward elimination logistic regression analysis was performed to find the significant variables (risk factors). The response variable (ESRD) in this study is binary; therefore, logistic regression analysis is used to identify the significant variables. We used a statistical package GLIM for fitting the model. For selection of initial model, the variables D, H, G, S, O, K, M, Hd, SLE, A, Ht, Hp & Ed, were used. Various factors which are significant at 5% level of significance are D, H, G, Ht and the interaction terms are: D*H, D*G, G*H and D*G*H.

To choose initial model, we used Brown method, for the backward elimination procedure. The suitable statistical technique to choose the risk factors of ESRD is logistic regression technique because the response variable, ESRD is a binary variable.

## Results

### Model for Both Sexes

For selection of initial model, the predictor variables D, H, G, S, O, Pk, M, Hd, SLE, A, Ht, Hp and Dr, were used. Various factors which are significant at 5% level of significance are D, H, G, Ht and the interaction terms are: (D*H), (D*G), (G*H) and (D*G*H). To get best possible fitted logistic model by using backward elimination procedure, we used SPSS. We take start from the model [D, H, G, Ht, (D*H), (D*G), (G*H) and (D*G*H)] for backward elimination procedure. Table 1 show the different steps used, with the coefficients, Standard Error (S.E), Wald Statistic and significance of each variable selected in the 6^th^ step of backward elimination procedure.

**Table 1:** Variables in the Equation.

	Variable	β	S.E.	Wald	df	Sig.	Exp(β)
Step 1	D	-2.488	0.575	18.734	1	0.000	0.083

	H	-1.457	0.673	4.683	1	0.030	0.233

	G	-1.383	0.536	6.650	1	0.010	0.251

	Ht	-0.056	0.368	0.023	1	0.879	0.946

	D * H	-0.147	0.856	0.030	1	0.864	0.863

	G * H	-1.104	0.874	1.597	1	0.206	0.332

	D * G	0.018	0.722	0.001	1	0.980	1.018

	D * G * H	1.382	1.174	1.386	1	0.239	3.982

	Constant	2.421	0.544	19.832	1	0.000	11.262

Step 2	D	-2.476	0.349	50.335	1	0.000	0.084

	H	-1.450	0.602	5.807	1	0.016	0.235

	G	-1.373	0.360	14.531	1	0.000	0.253

	Ht	-0.056	0.368	0.023	1	0.880	0.946

	D * H	-0.158	0.724	0.048	1	0.827	0.854

	G * H	-1.114	0.778	2.048	1	0.152	0.328

	D * G * H	1.400	0.926	2.285	1	0.131	4.054

	Constant	2.414	0.448	29.087	1	0.000	11.176

Step 3	D	-2.481	0.348	50.837	1	0.000	0.084

	H	-1.457	0.600	5.895	1	0.015	0.233

	G	-1.378	0.359	14.731	1	0.000	0.252

	D * H	-0.153	0.723	0.045	1	0.832	0.858

	G * H	-1.107	0.777	2.031	1	0.154	0.330

	D * G * H	1.393	0.925	2.269	1	0.132	4.028

	Constant	2.373	0.356	44.490	1	0.000	10.728

Step 4	D	-2.517	0.305	67.941	1	0.000	0.081

	H	-1.549	0.412	14.137	1	0.000	0.213

	G	-1.389	0.357	15.117	1	0.000	0.249

	G * H	-1.028	0.680	2.288	1	0.130	0.358

	D * G * H	1.276	0.740	2.977	1	0.084	3.582

	Constant	2.397	0.340	49.755	1	0.000	10.988

Step 5	D	-2.467	0.309	63.569	1	0.000	0.085

	H	-1.956	0.323	36.678	1	0.000	0.141

	G	-1.659	0.317	27.420	1	0.000	0.190

	D * G * H	0.729	0.627	1.350	1	0.245	2.073

	Constant	2.545	0.335	57.546	1	0.000	12.740

Step 6	D	-2.325	0.276	71.157	1	0.000	0.098

	H	-1.793	0.283	40.188	1	0.000	0.167

	G	-1.512	0.282	28.753	1	0.000	0.220

	Constant	2.385	0.296	64.901	1	0.000	10.857

Table 2 show the main effects (D, H & G) which are selected in the 6^th^ step of backward elimination along with the regression coefficient (β’s), p-value, odd ratios, Wald-statistic, standard error and the confidence interval for odd ratio. The p-values of diabetes, hypertension and glomerulonephritis (D, H and G) also indicate that these variables have significant contribution, since all values of p are less than 0.05. Finally, the logit model is given as:

**Table 2:** Variables in Equations.

Variable	β	S.E.	Wald	df	Sig.	Exp(B)	95% C.I. for Exp(β)	

							Lower	Upper
D	-2.325	0.276	71.157	1	0.000	0.098	0.057	0.168

H	-1.793	0.283	40.188	1	0.000	0.167	0.096	0.290

G	-1.512	0.282	28.753	1	0.000	0.220	0.127	0.383

Constant	2.385	0.296	64.901	1	0.000	10.857		

Logit (p^) = 2.385 – (2.325) D – (1.793) H – (1.512) G

### Model for Males

We take start from the model obtained by Brown method for backward elimination process for males, using SPSS. Table 3 shows the process of backward elimination procedure. Here we used initial model [D, H, G, Hd, (D*H, D*G), (H*G), (D*G*H)] obtained by using Brown method for backward elimination procedure. We obtained the final model in 5^th^ step contains only main effect (D, H, G, Hd), which is highly significant since all calculated p-values are less than 0.05. The regression coefficients, standard error, Wald-statistic, odds ratio and confidence interval of odds ratio for the various terms selected in the last step of backward elimination are given in Table 4. Thus, the fitted model is:

**Table 3:** Variables in Equations.

Variable		Β	S.E.	Wald	df	Sig.	Exp(β)
Step 1	D	3.336	1.270	6.894	1	0.009	28.094

	H	2.425	1.230	3.889	1	0.049	11.306

	G	2.356	1.193	3.898	1	0.048	10.543

	Hd	4.298	1.196	12.908	1	0.000	73.569

	D * H	-0.402	1.402	0.082	1	0.774	0.669

	D * G	-0.388	1.429	0.074	1	0.786	0.678

	G * H	-0.613	1.362	0.203	1	0.653	0.542

	D * G * H	18.775	6887.568	0.000	1	0.998	1.426E8

	Constant	-4.347	1.128	14.846	1	0.000	0.013

Step 2	D	3.051	0.679	20.191	1	0.000	21.130

	H	2.212	0.896	6.089	1	0.014	9.134

	G	2.092	0.649	10.390	1	0.001	8.098

	Hd	4.177	1.061	15.509	1	0.000	65.187

	D * H	-0.120	0.914	0.017	1	0.896	0.887

	G * H	-0.352	0.933	0.142	1	0.706	0.704

	D * G * H	18.390	6889.705	0.000	1	0.998	9.698E7

	Constant	-4.131	0.740	31.132	1	0.000	0.016

Step 3	D	2.986	0.463	41.614	1	0.000	19.810

	H	2.123	0.586	13.110	1	0.000	8.358

	G	2.078	0.637	10.649	1	0.001	7.985

	Hd	4.139	1.014	16.670	1	0.000	62.734

	G * H	-0.298	0.841	0.126	1	0.723	0.742

	D * G * H	18.334	6886.252	0.000	1	0.998	9.165E7

	Constant	-4.081	0.629	42.054	1	0.000	0.017

Step 4	D	3.032	0.448	45.842	1	0.000	20.740

	H	1.988	0.435	20.863	1	0.000	7.298

	G	1.924	0.458	17.677	1	0.000	6.846

	Hd	4.129	1.001	17.007	1	0.000	62.106

	D * G * H	18.211	6886.571	0.000	1	0.998	8.105E7

	Constant	-4.013	0.594	45.704	1	0.000	0.018

Step 5	D	3.245	0.435	55.527	1	0.000	25.651

	H	2.163	0.427	25.723	1	0.000	8.701

	G	2.164	0.435	24.763	1	0.000	8.702

	Hd	4.328	1.020	17.987	1	0.000	75.774

	Constant	-4.304	0.579	55.169	1	0.000	0.014

**Table 4:** Estimates of the Model Parameters.

Variable	β	S.E.	Wald	Df	Sig.	Exp (B)	95% C.I. for Exp (β)	
	
							Lower	Upper
D	3.245	0.435	55.527	1	0.000	25.651	8.0234	44.239

H	2.163	0.427	25.723	1	0.000	8.701	1.002	17.672

G	2.164	0.435	24.763	1	0.000	8.702	2.452	16.292

Hd	4.328	1.020	17.987	1	0.000	75.774	49.489	110.37

Logit (p^) = - 4.304 + (3.245) D+ (2.163) H + (2.164) G + (4.328) Hd

### Model for Females

From Brown method we get initial model [D, H, G, Hd, (D*G), (D*H), (H*G), (D*H*G)] and by using backward elimination procedure, we obtained the best fitted model with variables (H, D*H) in the 7^th^ step. Table 5 contains the variables H, (D*H), which are highly significant because calculated p-values are less than 0.05. The table consists of regression coefficients, Wald-statistic, standard error and odd ratios. Table 6 gives the information about various statistics of fitted model.

**Table 5:** Variables in Equations.

Variable		Β	S.E.	Wald	df	Sig.	Exp(β)
Step 1	D	0.369	1.235	0.089	1	0.765	1.447

	H	1.530	0.813	3.536	1	0.060	4.617

	G	-18.430	10620.802	0.000	1	0.999	0.000

	Hd	1.341	0.779	2.963	1	0.085	3.824

	D * H	1.968	1.384	2.022	1	0.155	7.156

	D * G	21.688	10620.802	0.000	1	0.998	2.623E9

	G * H	19.482	10620.802	0.000	1	0.999	2.891E8

	D * G * H	-22.238	10620.802	0.000	1	0.998	0.000

	Constant	-2.934	0.670	19.169	1	0.000	0.053

Step 2	H	1.442	0.745	3.747	1	0.053	4.230

	G	-18.518	10629.896	0.000	1	0.999	0.000

	Hd	1.293	0.754	2.945	1	0.086	3.645

	D * H	2.328	0.686	11.502	1	0.001	10.258

	D * G	22.048	10629.896	0.000	1	0.998	3.762E9

	G * H	19.567	10629.896	0.000	1	0.999	3.147E8

	D * G * H	-22.595	10629.896	0.000	1	0.998	0.000

	Constant	-2.837	0.566	25.158	1	0.000	0.059

Step 3	H	1.665	0.742	5.032	1	0.025	5.284

	Hd	1.328	0.754	3.106	1	0.078	3.774

	D * H	2.335	0.687	11.538	1	0.001	10.327

	D * G	3.760	1.347	7.795	1	0.005	42.939

	G * H	1.052	0.779	1.821	1	0.177	2.862

	D * G * H	-4.309	1.691	6.491	1	0.011	0.013

	Constant	-3.067	0.560	29.991	1	0.000	0.047

Step 4	H	2.106	0.646	10.619	1	0.001	8.211

	Hd	1.296	0.741	3.056	1	0.080	3.653

	D * H	1.886	0.574	10.799	1	0.001	6.590

	D * G	3.751	1.345	7.772	1	0.005	42.557

	D * G * H	-3.249	1.492	4.740	1	0.029	0.039

	Constant	-3.058	0.557	30.131	1	0.000	0.047

Step 5	H	2.081	0.632	10.843	1	0.001	8.011

	D * H	1.703	0.554	9.443	1	0.002	5.489

	D * G	3.512	1.329	6.987	1	0.008	33.500

	D * G * H	-3.042	1.478	4.234	1	0.040	0.048

	Constant	-2.818	0.515	29.983	1	0.000	0.060

Step 6	H	1.758	0.566	9.656	1	0.002	5.803

	D * H	1.471	0.532	7.655	1	0.006	4.355

	D * G	1.096	0.630	3.025	1	0.082	2.993

	Constant	-2.496	0.431	33.529	1	0.000	0.082

Step 7	H	1.690	0.562	9.047	1	0.003	5.420

	D * H	1.910	0.485	15.521	1	0.000	6.755

	Constant	-2.428	0.426	32.496	1	0.000	0.088

**Table 6:** Estimates of the Model Parameters.

Variable		β	S.E.	Wald	df	Sig.	Exp(B)	95% C.I. for Exp(β)	
	
								Lower	Upper
Step 1	H	1.69	0.562	9.047	1	0.003	5.42	1.802	16.305

	D * H	1.91	0.485	15.521	1	0.000	6.755	2.612	17.474

	Constant	-2.428	0.426	32.496	1	0.000	0.088		

Finally the selected model is:

Logit (p^) = -2.428 + (1.69) H + (1.91) D*H

## Discussion

ESRD is the last stage of the chronic renal failure in which kidneys become completely fail to function. In this stage, the kidney stops to remove the impurities and control electrolytes. The symptoms of ESRD comprise less urine output, swelling of legs and face, along with nausea vomiting. The basic aim of this study was to determine the important risk factors of ESRD and to construct a model for prediction of the ESRD patients. In this study, the response variable ESRD is a binary variable (equal to 1 if patient is ESRD and 0 if patient is non-ESRD), logistics regression analysis is, therefore, appropriate technique. At first we selected the initial model by Brown method. The initial model consists of the variables: D, H, G, Ht, (D * H), (D * G), (G * H) and

(D*G*H). Then we applied logistic regression to the data of all patients and through backward elimination procedure, we find the best fitted model for the whole date-set which contains three variables: D, H and G. It indicates that diabetic, hypertension and glomerulonephritis were significant risk factors of ESRD, where other predictor variables were turned out to be insignificant.

Next, we fitted logistic regression model for males and females data-set separately. For males, the predictor variables in the fitted model were D, H, G and Hd. This shows that diabetes, hypertension, glomerulonephritis and heredity were the main causes of ESRD. On the other hand, for the females, the predictor variables were H and (D*H), which shows that hypertension and diabetic hypertensive are significant factors of ESRD.

Our main conclusion from this analysis is that diabetes, hypertension and glomerulonephritis are the significant risk factors of ESRD and these factors play significant role in End Stage Renal Failure.

Our findings are in line with Kamal and Sakhuja [7] who suggested that hypertension, glomerulonephritis and diabetes are the main causes of ESRD in Pakistan and India.
